# Patient‐reported outcomes following autologous stem cell transplant for patients with multiple myeloma

**DOI:** 10.1002/jha2.231

**Published:** 2021-06-27

**Authors:** Noa Biran, Wanting Zhai, Roxanne E. Jensen, Jeanne Mandelblatt, Susan Kumka, Rashmi Unawane, Kristi D. Graves, David H Vesole, David S Siegel, Arnold L. Potosky

**Affiliations:** ^1^ Hackensack Meridian Health John Theurer Cancer Center Hackensack New Jersey USA; ^2^ Cancer Prevention and Control Program, Lombardi Comprehensive Cancer Center Georgetown University Medical Center Washington District of Columbia USA; ^3^ Outcomes Research Branch, Healthcare Delivery Research Program National Cancer Institute Bethesda Maryland USA

**Keywords:** autologous stem cell transplant, cognitive function, multiple myeloma, patient‐reported outcomes, quality of life

## Abstract

We evaluated changes in patient‐reported outcomes and cognitive function from pre‐ to 3–6 months post‐treatment among 42 newly diagnosed patients with multiple myeloma undergoing transplant with complete data using PROMIS‐29. There were statistically significant improvements in physical (*p* < .001) and mental health (*p* < .001) but not cognition from pre‐treatment to 3–6 month follow‐up. Similar results were seen within age or comorbidity strata. Patients with myeloma undergoing transplant experienced generally improved short‐term health outcomes with no significant declines in cognition.

## 1 INTRODUCTION

In 2021, over 34,000 new cases of multiple myeloma and nearly 12,400 deaths are projected to occur. With recent advances in treatment, overall 5‐year survival in patients with multiple myeloma is now 56% with most getting a transplant [1]. The standard of care for transplant‐eligible patients is induction therapy followed by consolidative high‐dose therapy with autologous stem cell transplantation (ASCT). The intensity and duration of treatment may cause severe short‐term treatment toxicities, long‐term symptoms, and a decline in physical and cognitive functions, especially among older patients [2, 3, 4, 5, 6]. With recent therapeutic advances extending survival, but increasing toxicity, evaluating how these treatments affect short‐term patients will help promote informed decisions about treatment and identify areas for additional clinical support and intervention.

We prospectively evaluated multiple domains of patient‐reported outcomes among patients diagnosed with myeloma. Our primary aim was to describe pre‐ and post‐transplant changes in terms of symptoms, functional deficits, and cognitive function in patients with multiple myeloma undergoing autologous stem cell transplant.

## 2 METHODS

## 2.1 Participants

We enrolled patients with newly diagnosed symptomatic myeloma, both transplant eligible and ineligible, who had received or completed at least one cycle of induction therapy at the Hackensack John Theurer Cancer Center (JTCC) or the Georgetown University Lombardi Comprehensive Cancer Center (LCCC) consortium. Additional eligibility criteria included the ability to provide informed consent and read and speak English. Consecutive patients seen during office hours were identified, screened, and invited to participate until enrollment was complete. Patients were excluded if there was a predetermined plan for tandem ASCT. The study was approved by both institution's Institutional Review Boards and all participants provided informed consent.

### 2.2 Procedures

Patients were recruited from October 2016 to February 2019 and were on average 4‐months postdiagnosis. The first survey (T0) was given at the time of, or just prior to, induction therapy, but before ASCT, if ASCT was received. Follow‐up assessments occurred at the first post‐ASCT visit (T1), and approximately 1‐month (T2), 3‐ and 6‐months post‐ASCT (T3 and T4, respectively). Participants who did not receive ASCT were followed at corresponding visits. Assessments were conducted either in person or using a secure, web‐based application. A phone‐based option was also available. All objective cognitive assessments were performed in person. Participants received a $20 gift card after the baseline assessment and another at completion of study assessments.

### 2.3 Measures

#### 2.3.1 Primary outcomes

Five symptoms (fatigue, pain, depression, anxiety, and sleep disturbance) and two functional domains (physical and social function) were assessed using the patient‐reported outcomes measurement information system (PROMIS^®^) Profile‐29. This profile includes separate domain scores plus summary scores for physical and mental health. We also included the PROMIS cognitive function domain (Cognitive Problems 4a). All PROMIS scores are transformed on a T‐score metric against the U.S. average population (0–100 scale with mean = 50, SD = 10 points). Higher symptom scores and lower functioning scores reflect poorer quality of life. Changes of 3 to 6 points on these scores are defined as clinically meaningful changes [7]. We used the 11‐item comprehensive score for financial toxicity (COST) scale (ranging from 4 to 34) to quantify cancer patients’ experience of financial distress [8]. We also asked patients about selected comorbid conditions.

#### 2.3.2 Secondary outcomes and neuropsychological assessment

We used a validated battery of cognitive functioning tests to assess neuropsychological function. At T0 or T1 and T4, a trained research assistant administered the neurophysiological assessment in two selected domains: attention, processing speed, and executive function (APE); and learning and memory (LM) [9, 10].

#### 2.3.3 Covariates

We abstracted information from institutional electronic medical records to ascertain tumor‐, treatment‐, and transplant‐related information, disease staging, and use of prior therapies. The survey was used to collect information on patient's sex, age, race, ethnicity, and comorbidity.

### 2.4 Analysis

We calculated proportions, means, and standard deviations for all variables at each time point. We conducted bivariate analysis for continuous PROMIS T‐scores reflecting symptom and functional outcomes using *t*‐tests (or Wilcoxon Rank sum tests), and χ^2^ tests (or Fisher's exact test) for categorical or dichotomous outcomes, respectively. We used *p* < .05 to define statistical significance.

## 3 RESULTS

There were 93 eligible patients, and of those 78 (84%) agreed to participate, 8 of these patients were later deemed ineligible. Of the remaining 70 patients, 53 (76%) received ASCT and 17 (24%) did not within 12 months of diagnosis. The only statistically significant differences observed between the two groups were for stage and first‐line therapy (Table 1). ASCT recipients were more likely to have international stage (ISS) 1 disease (56 vs. 29%), and more frequently received first‐line induction therapy with bortezomib (21 vs. 6%) or carfilzomib‐based (68 vs. 59%) triplet therapy compared to non‐ASCT patients.

**TABLE 1:** Characteristics of study cohort of newly diagnosed multiple myeloma patients from a single consortium

		Received ASCT	
	All	No	Yes
**Total (*N*)**	70	17	53
**Sex**			
Female	60.0	47.1	64.2
Male	40.0	52.9	35.8
**Age at diagnosis**			
Less than 65	41.4	29.4	45.3
65 or greater	58.6	70.6	54.7
**Race/ethnicity**			
White non‐Hispanic	62.9	41.2	69.8
Other	34.3	52.9	28.3
**Comorbid conditions**			
None	41.4	41.2	41.5
One	18.6	23.5	17.0
Two or more	40.0	35.3	41.5
**ECOG performance status at baseline (self‐reported)**			
Fully active	15.7	23.5	13.2
Restricted in physically strenuous activity	61.4	58.8	62.3
Require bed rest less than 50% of the waking day	11.4	17.6	9.4
Require bed rest more than 50% of the waking day	10.0	0.0	13.2
**International stage system^a^**			
Stage 1 or 2	50.0	29.4	56.6
Stage 3	28.6	58.8	18.9
Missing	21.4	11.8	24.5
**First‐line treatment^a^**			
Bortezomib (velcade)‐based triplet	17.1	5.9	20.8
Carfilzomib‐based triplet	65.7	58.8	67.9
Other	11.4	29.4	5.7

*Note* ^:^ Some percentages do not total 100% due to missing values.

^a^Differences between non‐ASCT and ASCT groups were statistically significant (Fischer's exact test *p* < .05) for these variables.

From the 70 enrolled participants, 20 were either lost to follow‐up or died. Among the 50 remaining patients, only 8 did not receive ASCT. When we compared the 42 ASCT cases to 8 non‐ASCT cases with respect to changes in any patient‐reported outcomes and the neuropsychological assessment from baseline to the last assessment, we observed no statistically significant differences (data not shown). Due to the small sample of non‐ASCT cases, we focused the analysis on the remaining 42 ASCT cases.

### 3.1 Short‐term outcomes

Among the 42 ASCT subjects, we observed statistically and clinically meaningful improvements in overall physical health (+5.9, *p* < .001) and mental health (+4.4, *p* < .001) from T0, before ASCT to T4, 6 months post‐ASCT (Table 2). Improvements were reflected by changes in the physical and mental health summary domains, such as improved physical function scores, decline in physical symptoms (pain, fatigue), and decline in mental health symptoms (anxiety, depression). Changes in these individual domains were 3 points or greater, reflecting moderate to large effect sizes [11].

**TABLE 2:** Changes in patient‐reported outcome symptom and functioning domain scores at baseline (T0) and the last assessment^*^ among ASCT recipients (*n* = 42)

	Baseline (T0) assessment mean scores(95% CI)	Last assessment mean scores^a^(95% CI)	Comparison of mean scores(*p*‐value)^b^
PROMIS‐29 V2.0 summary			
Physical health score	41.2 (38.1,44.2)	47.1 (44.2,50.1)	<.001
Mental health score	46.4 (43.8,49.0)	50.8 (48.2,53.3)	<.001
PROMIS domains: Symptoms			
Anxiety	53.3 (49.9,56.7)	49.2 (46.1,52.3)	<.01
Depression	49.2 (46.0,52.3)	46.0 (43.4,48.6)	<.01
Fatigue	54.2 (51.0,57.5)	49.1 (45.9,52.4)	<.01
Pain interference	57.2 (53.3,61.2)	51.6 (48.8,54.3)	<.01
Sleep disturbance	52.0 (50.5,53.4)	53.0 (51.5,54.5)	0.35
PROMIS domains: Function			
Ability to participate in social roles and activities	47.9 (44.4,51.5)	52.1 (48.7,55.5)	0.02
Physical function	41.0 (38.1,43.9)	46.7 (43.8,49.6)	<.001
Cognitive function	52.8 (49.7,56.0)	51.8 (48.8,54.7)	0.40
Other domain			
Financial distress (COST scale)^c^	28.2 (26.0,30.4)	27.9 (25.6,30.2)	0.77

*Note*: All PROMIS domain scores are reported as t‐scores on a 0–100 scale (with mean = 50 and SD = 10 for the general U.S. population. More of the latent trait is reflected in higher score.

^a^For 12 of the 42 cases, T3 (3‐month follow‐up) was used as the last assessment.

^b^ *p*‐values calculated from the *t*‐test of two means.

^c^Change in financial distress is based on change in score on the comprehensive assessment of financial toxicity (COST) scale. Lower scores represent worse financial distress.

There was no significant change observed in cognitive function over time among the ASCT recipients. In stratified analyses, we observed similar patterns, meaning no significant change in cognitive function, according to age group (age 65 and older) and comorbidity (any vs. none). We next assessed the changes in cognition based on performance on the cognitive battery of tests. Among the 17 ASCT cases tested, APE scores significantly improved by +0.40 (SD 0.47) (*p* = 0.003). The change in LM scores increased by 0.007 (SD 0.45, *p* = 0.95).

## 4 DISCUSSION

Results from this preliminary study suggest that patients with myeloma who receive ASCT experience either improvements or no change in several patient‐reported outcomes. As expected, at baseline (T0), patients reported moderate to severe impairments in physical function, pain, and fatigue. All of these improved or resolved by the final assessment. Importantly, we observed overall improvement or resolution of impairments in physical function, pain, and fatigue among patients aged 65 or older and in those with comorbidities. These results are consistent with other recent prospective studies in patients with multiple myeloma who reported low levels of impairment in memory, motor speed, and attention pretreatment and post‐ASCT [12, 13, 14]. Prior studies have demonstrated that although ASCT leads to short‐term deterioration in quality of life and symptom burden in patients with multiple myeloma, the adverse impact is short‐lived with a return to baseline health status as early as 1–2 months post‐ASCT [4]. If replicated in larger studies, our findings may have implications in practice, particularly when applied to patients over the age of 65. Current practice patterns indicate that ∼80% of older patients do not receive ASCT treatment [15]. Results from our pilot study suggest that patients who receive ASCT appear to tolerate this intensive regimen as reflected by recovery of good post‐treatment function.

Strengths of our study included our use of state‐of‐the‐art, well validated PRO measures and our collection of data prior to ASCT. The main limitation of our study was the small sample size, with very few non‐ASCT patients to compare with ASCT patients over time. This represents an important limitation of our single‐consortium study where the majority of patients with myeloma were deemed transplant eligible and went on to receive ASCT in the first year from initial diagnosis. This is also a single institution study, and will need replications in other settings.

Our work contributes to the growing literature that examines patient‐reported outcomes among patients with myeloma undergoing ASCT, with indication that patients retain baseline functioning. Future research is needed to measure patient‐reported outcomes in larger, longitudinal prospective observational studies.

## AUTHOR CONTRIBUTION STATEMENTS

NB, AP and RJ conceived and designed the work, contributed to data collection, data analysis, and interpretation, and drafting the article. WZ contributed to data analysis and critical revision of the article. JM contributed to conception of the work, data interpretation, critical revision and final approval of the manuscript. SK and RU contributed to data collection and critical revision of the article. KG contributed to data analysis and interpretation, drafting the manuscript, critical revision of the article, and final approval of the version to be published. DHV contributed to data interpretation, critical revision and final approval of the manuscript. DSS contributed to conception of the work, data interpretation, critical revision of the article and final approval.

## CONFLICTS OF INTEREST

The article was prepared as part of one of the author's (REJ) official duties as employees of the US Federal Government. The findings and conclusions in this report are those of the authors and do not necessarily represent the official position of the National Cancer Institute. The authors have no conflicts of interest to disclose.
